# Fractional flow reserve measurements and long-term mortality—results from the FLORIDA study

**DOI:** 10.3389/fcvm.2024.1337941

**Published:** 2024-02-09

**Authors:** Felicitas Boeckling, Barbara E. Stähli, Tanja Rudolph, Matthias Lutz, Anne-Sophie Schatz, Tobias Vogelmann, Magnus Stueve, Nick E. J. West, Els Boone, Aslihan Erbay, David M. Leistner

**Affiliations:** ^1^ Department of Medicine, Cardiology, University Hospital Frankfurt, Goethe University, Frankfurt, Germany; ^2^ Department of Cardiology, University Heart Center, University Hospital Zurich, Zurich, Switzerland; ^3^ Department for General and Interventional Cardiology/Angiology, Heart- und Diabetes Center NRW, Ruhr-Universität Bochum, Bad Oeynhausen, Germany; ^4^ Department of Cardiology and Angiology, University Hospital Schleswig-Holstein, Kiel, Germany; ^5^ Department of Cardiology, Charité—University Medicine Berlin, Berlin, Germany; ^6^ LinkCare GmbH, Stuttgart, Germany; ^7^ Abbott Vascular, Santa Clara, CA, United States

**Keywords:** fractional flow reserve, acute coronary syndrome, chronic coronary syndrome, percutaneous coronary intervention, mortality, real-world evidence

## Abstract

**Background:**

Randomized evidence suggested improved outcomes in fractional flow reserve (FFR) guidance of coronary revascularization compared to medical therapy in well-defined patient cohorts. However, the impact of FFR-guided revascularization on long-term outcomes of unselected patients with chronic or acute coronary syndromes (ACS) is unknown.

**Aims:**

The FLORIDA (Fractional FLOw Reserve In cardiovascular DiseAses) study sought to investigate outcomes of FFR-guided vs. angiography-guided treatment strategies in a large, real-world cohort.

**Methods:**

This study included patients enrolled into the German InGef Research Database. Patients undergoing coronary angiography between January 2014 and December 2015 were included in the analysis. Eligible patients had at least one inpatient coronary angiogram for suspected coronary artery disease between January 2014 and December 2015. Patients were stratified into FFR arm if a coronary angiography with adjunctive FFR measurement was performed, otherwise into the angiography-only arm. Matching was applied to ensure a balanced distribution of baseline characteristics in the study cohort. Patients were followed for 3 years after index date and primary endpoint was all-cause mortality.

**Results:**

In the matched population, mortality at 3 years was 9.6% in the FFR-assessed group and 12.6% in the angiography-only group (*p* = 0.002), corresponding to a 24% relative risk reduction with use of FFR. This effect was most pronounced in patients in whom revascularization was deferred based on FFR (8.7% vs. 12.3%, *p* = 0.04) and in high-risk subgroups including patients aged ≥75 years (14.9% vs. 20.1%, *p* < 0.01) and those presenting with ACS (10.2% vs. 14.0%, *p* = 0.04).

**Conclusions:**

FFR-based revascularization strategy was associated with reduced mortality at 3 years. These findings further support the use of FFR in everyday clinical practice.

## Introduction

Fractional flow reserve (FFR) has been established as a valuable tool for functional assessment of intermediate coronary lesions, with proven safety and efficacy when compared with angiography alone (1–3). Current guideline-based (4, 5) approach to such lesions recommends functional assessment based on the growing evidence derived from numerous randomized trials demonstrating superiority of FFR-guided percutaneous coronary intervention (PCI) over use of coronary angiography alone, predominantly in patients with chronic coronary syndrome (CCS) (6, 7). A recent meta-analysis including 2,400 patients of very selected patient cohorts from the three available randomized trials to date has reported a 28% reduction of the primary endpoint, a composite of cardiac death or myocardial infarction, with an FFR-based coronary revascularization strategy compared with optimal medical therapy (OMT), and differences were mainly driven by reduced rates of myocardial infarction in the FFR group (8). These studies, however, failed to show any mortality benefit for FFR-guided PCI in comparison with angiography-based coronary revascularization (9). Recently, two larger observational studies have reported for the first time a mortality benefit of FFR-based strategies for coronary revascularization (10, 11), although these studies were limited by the restriction to selected patient subgroups.

Therefore, the objective of the present study is to assess the effect of an FFR-guided compared to an angiography-guided revascularization strategy on mortality in a large, real-world patient cohort, including patients with different stages of coronary artery disease as well as patients with acute coronary syndrome (ACS).

## Methods

### Dataset and study sample

The FLORIDA study (Fractional FLOw Reserve In cardiovascular DiseAses) is a population-based, observational cohort study based on the prospective German InGef (Institute for Applied Health Research Berlin, Berlin, Germany) Research Database.

The InGef Research Database (formerly: Health Risk Institute) is an anonymized German health claims data base comprising longitudinal data from over 6 million people insured in one of 70 statutory German health insurances contributing data to the database (12, 13). For the purpose of this analysis, the dataset was adjusted by age and sex to match the demographic structure of the German population and condensed to a sample of 4,395,540 people (14). The InGef Research Database contains demographic data as well as information on hospitalization, main diagnosis, secondary diagnoses, diagnostic and therapeutic procedures, outpatient physician visits, and outpatient drug prescriptions. Diagnoses are coded according to the German Modification of the International Classification of Diseases, 10th Revision (ICD-10-GM).

The study design was predefined in a detailed study protocol and the study registered at clinicaltrials.gov (NCT04597489).

### Study population

**Figure 1:**
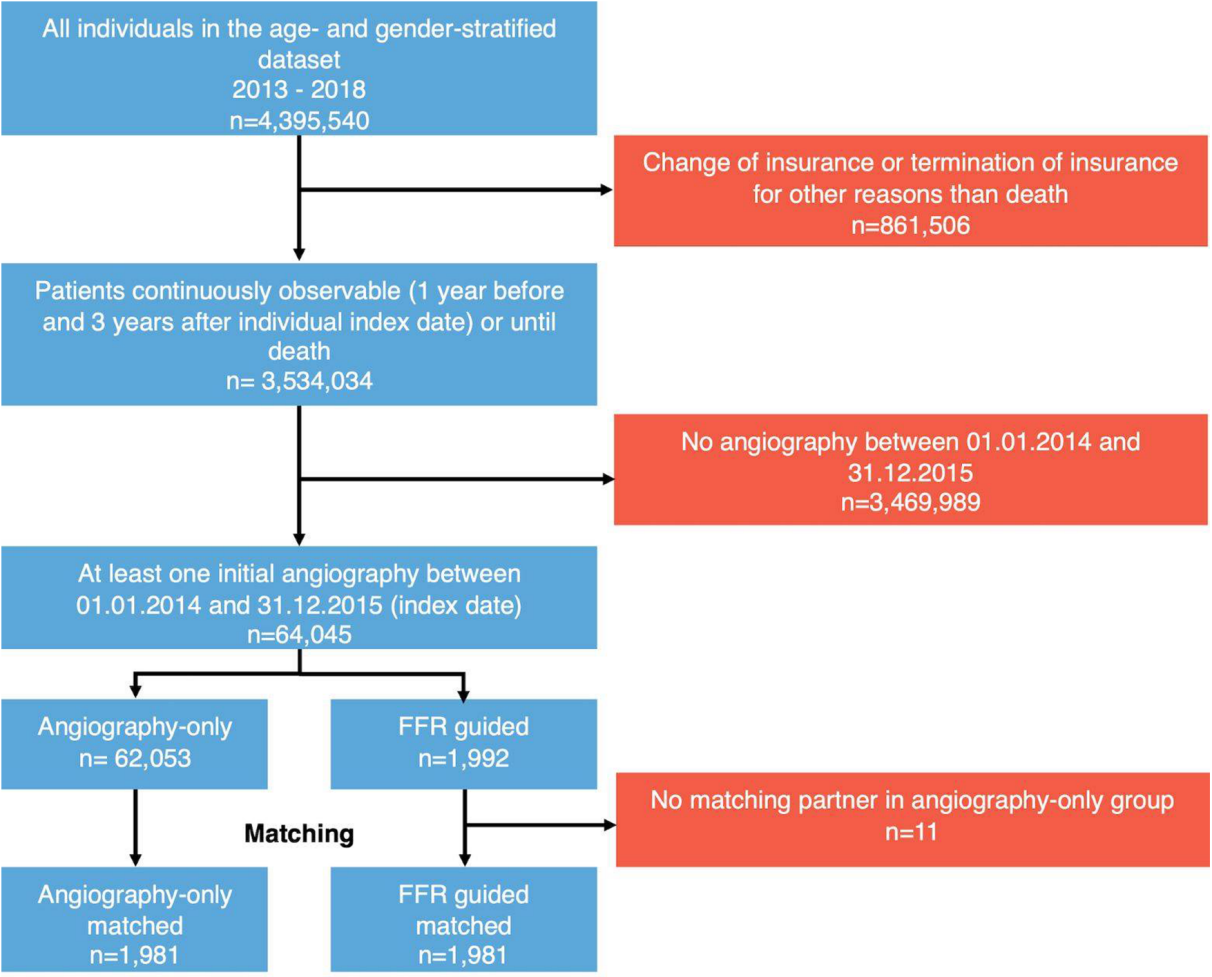
Study flow diagram of the FLORIDA study. Patients with at least one within-hospital angiography were included. Patients were matched for sex, presence of acute coronary syndrome, age ± 5 years, and propensity scores, with each FFR patient matched to the closest angiography-only patient (for details: see text). FFR, fractional flow reserve.

Out of a dataset of 4,395,540 age- and sex-stratified individuals in the German InGef Database, 64,045 patients that underwent at least one inpatient coronary angiogram for suspected coronary artery disease between January 2014 and December 2015 were included in the analysis (Figure 1). The discharge date of the first hospital stay involving a coronary angiogram was defined as the patient's individual index date. Patients were included into the study population regardless of their underlying disease (all-comers approach), if they were observable for at least 4 years (1 year prior the time of inclusion and 3 years after the time of inclusion), or died within follow-up.

### Study procedures

Patients were stratified into the FFR group if a coronary angiogram with adjunctive FFR measurement was performed during the index hospitalization; patients were stratified into the angiography-only group if a coronary angiogram without adjunctive FFR measurement was performed.

### Patient characteristics, index diagnosis, and index treatments

Baseline characteristics, medical treatments, and comorbidities including prior events were determined using inpatient and outpatient health claims for the 365 days prior to the index date. Patients were stratified by index diagnoses, i.e., ACS or CCS according to the index hospital admission diagnosis and further by the type of treatment received, i.e., revascularization by PCI or coronary artery bypass grafting (CABG), or conservative management with OMT alone, based on the procedure codes during the index hospital.

### Risk adjustment

To minimize selection bias, a nearest-neighbor matching approach was used to create a comparable data set of patients undergoing FFR-based and those undergoing angiography-based treatment strategy. Risk adjustment was performed with a mix of direct matching and propensity score (PS) matching to effectively reduce potential confounders (15) and PS was estimated by logistic regression (logit model) including use of FFR as the dependent variable. Based on prior studies, cardiovascular risk factors were included as independent variables (6, 16). In total, 72 variables were used for PS estimation including chronic heart failure, prior cardiovascular events (myocardial infarction and stroke), procedures during index hospitalization (PCI, and CABG), pre-existing cardiovascular risk factors including diabetes and hypertension, cancer diagnosis, previous medical cardiovascular therapy (e.g., betablockers, angiotensin converting enzyme inhibitors/angiotensin receptor blockers, and statins), as well as the 20 most common other comorbidities and medical treatments (see Supplementary Table S1 for all covariates used in PS estimation). Each independent variable was assessed 365 days prior to the index day. Finally, each FFR patient was matched on the day of angiography to an angiography-only patient with the same sex, age with a caliper of ±5 years, ACS or CCS admission diagnosis, and the closest estimated PS (estimated patient's PS were allowed to vary by ±0.2 * SD). The matching was performed as an individual 1:1 match between FFR and angiography-only patients without replacement.

### Follow-up and outcome assessment

All patients were followed for a period of 3 years after the index date or until death. The primary endpoint was mortality at 3 years, prospectively assessed on a quarterly basis.

### Statistical analysis

The cohorts' clinical characteristics were evaluated using standardized mean differences (SMD), defined as the absolute difference in means (or proportions) divided by the average standard deviation (SD). Differences in variables were assessed using chi-squared tests with Yates' correction for categorical variables and two-sample *t*-tests for continuous variables. The log-rank test was used to compare hazard rates for the two groups which were also presented as life-table curves. A two-sided *p*-value < 0.05 was considered to indicate statistical significance. Results from the logistic regression analyses were presented as odds ratios (OR) with 95% confidence intervals (CI). All data were analyzed using SAS® 9.4 (SAS Institute Inc., Cary, NC, USA) and R 3.4.0 (The R Foundation for Statistical Computing, Vienna, Austria).

## Results

### Study population

Of 64,045 patients analyzed, FFR was performed in 1,992 (3.11%) patients following angiography (Figure 1). 37.4% and 31.9% of patients underwent coronary angiography for ACS in the FFR and angiography-only groups, respectively (*p* < 0.001). Patients in the FFR group were older (*p* = 0.008), more frequently male (*p* < 0.001), were more likely to have had prior coronary artery disease (*p* < 0.001), and were more often on betablocker (*p* < 0.001) and statin therapy (*p* < 0.001, Table 1). A comprehensive comparison of the cohorts before matching (Supplementary Table S2) and after matching (Supplementary Table S3) can be found in the Supplemental Materials.

**Table 1:** Baseline and procedural characteristics.

	Total study cohort	FFR group	Angiography-only group	SMD in % (*p*-value)
*N*	64,045	1,992	62,053	–
Demographics				
Male gender	40,475 (63.2)	1,345 (67.5)	39,130 (63.1)	9.38 (<0.001)
Mean age (SD)	68.60 (11.02)	69.28 (10.29)	68.58 (11.65)	6.7 (0.008)
Age <65 years	22,026 (34.4)	628 (31.5)	21,398 (34.5)	−6.29 (0.007)
Age 65 ≤75 years	18,488 (28.9)	623 (31.3)	17,865 (28.8)	5.42 (0.017)
Age ≥75 years	23,531 (36.7)	741 (37.2)	22,790 (36.7)	0.98 (0.684)
Comorbidities/risk factors				
Known CAD	29,773 (46.5)	1,160 (58.2)	28,613 (46.1)	24.45 (<0.001)
Known heart failure	14,479 (22.6)	426 (21.4)	14,053 (22.6)	−3.04 (0.195)
Renal failure	990 (1.5)	37 (1.9)	953 (1.5)	2.49 (0.292)
Diabetes mellitus	21,124 (33.0)	645 (32.4)	20,479 (33.0)	−1.33 (0.577)
Arterial hypertension	49,509 (77.3)	1,653 (83.0)	47,856 (77.1)	14.71 (<0.001)
Obesity	14,853 (23.2)	481 (24.1)	14,372 (23.2)	2.32 (0.318)
Dyslipidemia	37,380 (58.4)	1,295 (65.0)	36,085 (58.2)	14.13 (<0.001)
Medical therapy				
Antiplatelets	14,633 (22.8)	588 (29.5)	14,045 (22.6)	15.73 (<0.001)
Beta blockers	30,944 (48.3)	1,122 (56.3)	29,822 (48.1)	16.61 (<0.001)
ACE inhibitors/ARB	21,604 (33.7)	706 (35.4)	20,898 (33.7)	3.71 (0.106)
Statins	25,752 (40.2)	1,019 (51.2)	24,733 (39.9)	22.83 (<0.001)
Procedural characteristics				
Angiography for				
ACS	23,960 (37.4)	635 (31.9)	23,325 (37.6)	−4.7 (<0.001)
CCS	40,085 (62.6)	1,357 (68.1)	38,728 (62.4)	4.7 (<0.001)
Revascularization by	23,659 (36.9)	867 (43.5)	22,792 (36.7)	13.89 (<0.001)
PCI	22,851 (35.7)	858 (43.1)	21,993 (35.4)	15.67 (<0.001)
CABG	808 (1.2)	9 (0.4)	799 (1.3)	−9.01 (0.001)

Values are *n* (%). Angiography-only group received angiography without FFR guidance. SMD is in %. ACE, angiotensin-converting-enzyme; ACS, acute coronary syndrome; CABG, coronary artery bypass graft; CAD, coronary artery disease; CCS, chronic coronary syndrome; FFR, fractional flow reserve; PCI, percutaneous coronary intervention; SD, standard deviation, and SMD, standardized mean difference.

### Matched study population

The estimation of PS for matching indicated a significant difference with respect to the covariates used in the PS estimate between the two groups (log-likelihood 416.1388, *p* < 0.0001). For regression results of the PS estimation, see Supplementary Table S1. After matching, the groups consisted of 1,981 patients each, including a subgroup of 37.1% of patients aged ≥75 years (*n* = 735 resp. 736) and a subgroup of 31.7% of patients with ACS as index event (*n* = 629). A total of 1,338 (67.5%) patients in both groups were male, and importantly, no other meaningful differences between groups were detected after matching (Table 2).

**Table 2:** Baseline and procedural characteristics after matching.

Variable	After matching			
	Total study cohort	FFR group	Angiography-only group	SMD in % (*p*-value)
*N*	3,962	1,981	1,981	–
Demographics				
Male gender	2,676 (67.5)	1,338 (67.5)	1,338 (67.5)	0 (>0.9999)
Mean age (SD)	69.31 (10.31)	69.31 (10.31)	69.31 (10.31)	0 (>0.9999)
Age <65 years	1,244 (31.4)	622 (31.4)	622 (31.4)	0 (>0.9999)
Age 65 ≤75 years	1,247 (31.5)	623 (31.4)	624 (31.5)	−0.11 (>0.9999)
Age ≥75 years	1,471 (37.1)	736 (37.2)	735 (37.1)	0.1 (>0.9999)
Comorbidities/risk factors				
Known CAD	2,301 (58.1)	1,149 (58.0)	1,152 (58.2)	−0.31 (0.9487)
Known heart failure	864 (21.8)	425 (21.5)	439 (22.2)	−1.71 (0.6170)
Renal failure	65 (1.6)	37 (1.9)	28 (1.4)	3.58 (0.3171)
Diabetes mellitus	1,273 (32.1)	643 (32.5)	630 (31.8)	1.41 (0.6831)
Arterial hypertension	3,271 (82.6)	1,642 (82.9)	1,629 (82.2)	1.73 (0.6154)
Obesity	938 (23.7)	478 (24.1)	460 (23.2)	2.14 (0.5252)
Dyslipidemia	2,625 (66.3)	1,287 (65.0)	1,338 (67.5)	−5.45 (0.0929)
Medical therapy				
Antiplatelets	1,162 (29.3)	578 (29.2)	584 (29.5)	−0.67 (0.8615)
Beta blockers	2,209 (55.8)	1,114 (56.2)	1,095 (55.3)	1.93 (0.5648)
ACE inhibitors/ARB	1,425 (36.0)	700 (35.3)	725 (36.6)	−2.63 (0.4269)
Statins	2,064 (52.1)	1,011 (51.0)	1,053 (53.2)	−4.24 (0.1923)
Procedural characteristics				
Angiography for				
ACS	1,258 (31.8)	629 (31.8)	629 (31.8)	0 (>0.9999)
CCS	2,704 (68.2)	1,352 (68.2)	1,352 (68.2)	0 (>0.9999)
Revascularization by	1,575 (39.8)	864 (43.6)	711 (35.9)	15.83 (<0.0001)
PCI	1,537 (38.8)	855 (43.1)	682 (34.4)	−100.12 (<0.0001)
CABG	38 (1.0)	9 (0.5)	29 (1.5)	115.70 (<0.0001)

Values are *n* (%). Angiography-only group received angiography without FFR guidance. SMD is in %. ACE, angiotensin-converting-enzyme; ACS, acute coronary syndrome; CABG, coronary artery bypass graft; CAD, coronary artery disease; CCS, chronic coronary syndrome; FFR, factional flow reserve; PCI, percutaneous coronary intervention; SD, standard deviation; and SMD, standardized mean difference.

**Figure 2:**
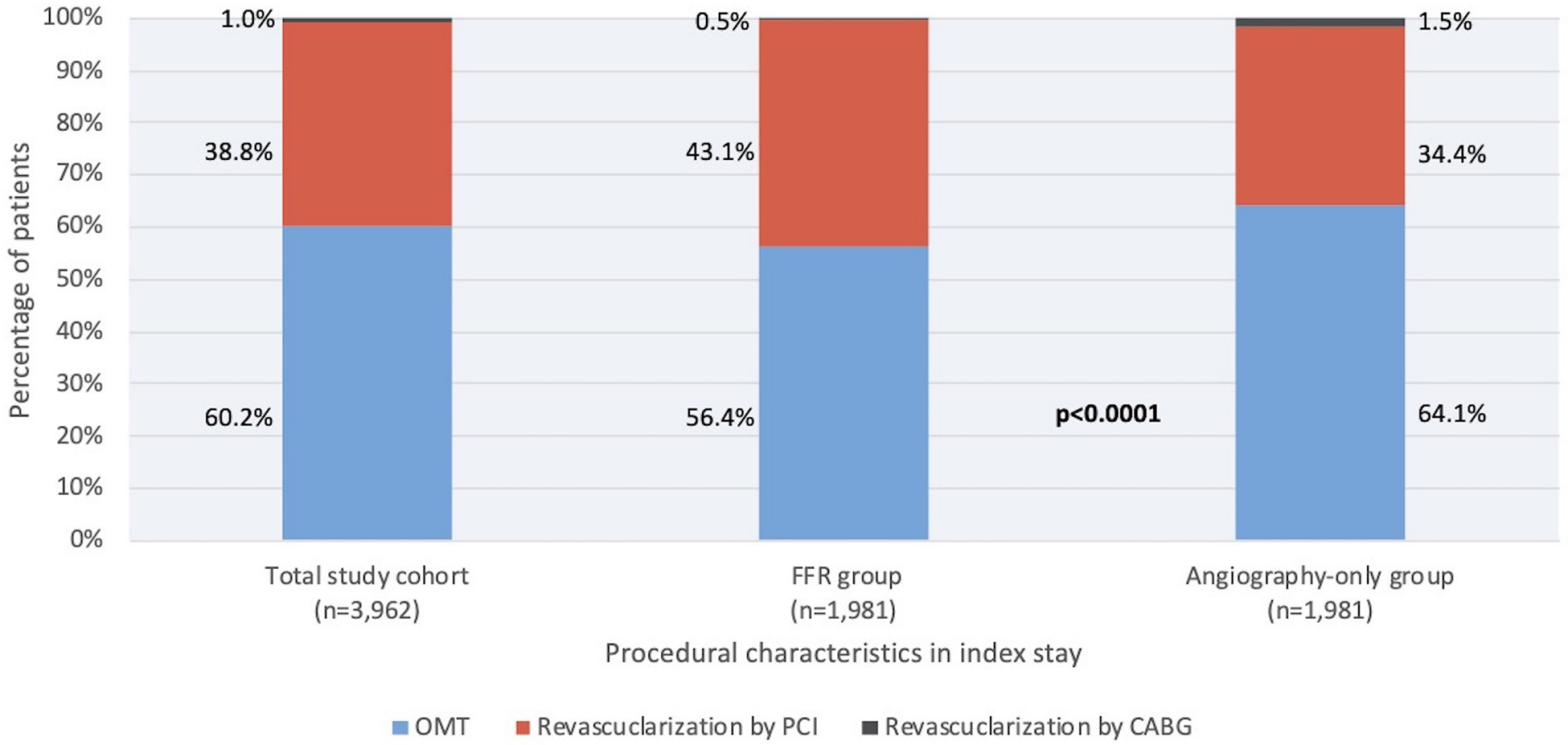
Treatment strategies in the total patient cohort, in patients undergoing FFR guidance, and in patients undergoing angiography guidance for PCI. Percentage of patients treated by PCI (blue) CABG (black), or optimal medical therapy (red) are shown. This proportion differed between patients evaluated by FFR (middle bar) and angiography-only (right bar), with a lower percentage of patients treated by PCI in the FFR group (*p* < 0.001). ABG, coronary artery bypass graft; FFR, fractional flow reserve; OMT, optimal medical therapy; PCI, percutaneous coronary intervention.

Coronary revascularization was performed in 864 (43.6%) patients in the FFR group and in 711 (35.9%) patients in the angiography-only group (*p* < 0.0001). In the FFR group, a total of 855 (43.1%) patients underwent PCI and 9 (0.5%) patients CABG; in the angiography-only group, a total of 682 (34.4%) patients underwent PCI and 29 (1.5%) patients CABG, respectively (Figure 2).

### FFR use and all-cause mortality

**Figure 3:**
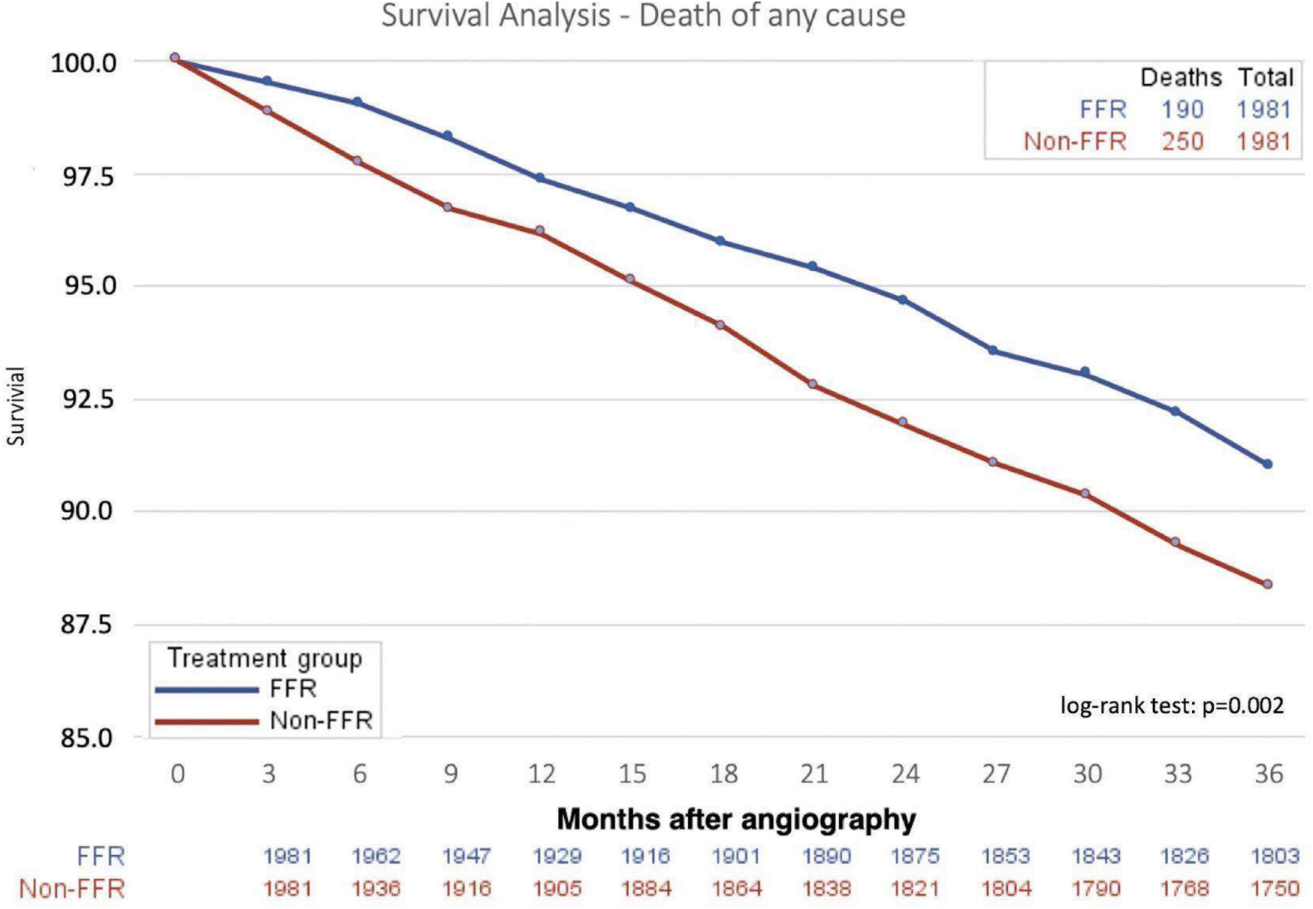
Survival in patients undergoing FFR or angiography guidance for coronary artery disease therapy. FFR, fractional flow reserve.

Overall mortality was 4.1% at 1 year, 7.7% at 2 years and 11.1% at final 3 year follow-up. Three-year mortality was 9.6% in the FFR and 12.6% in the angiography-only group, respectively (*p* = 0.002, Table 3), corresponding to a 24% relative risk reduction and a number needed to treat (NNT) of 34 to prevent one death using FFR compared with angiography alone. Consistently, mortality rates at one (3.3% vs. 4.9%, *p* = 0.013) and 2 years (6.5% vs. 8.9%, *p* = 0.004) were lower in the FFR-guided treatment group compared with the angiography-only group (Figure 3, Table 3).

**Table 3:** Mortality rates within the study cohort as well as predefined subgroups over3-years of follow-up.

			Overall	Sub-group ACS	Subgroup CCS	≥75 years	Revascularization	
							Yes	No
Total study cohort	*N* (%)		3,962	1,258 (31.7)	2,704 (68.2)	1,471 (37.1)	1,575 (39.8)	2,387 (60.2)
	Cumulative mortality *N* (%)	1 year	162 (4.1)	54 (4.3)	108 (4.0)	109 (7.4)	67 (4.4)	95 (4.0)
		2 year	305 (7.7)	108 (8.6)	197 (7.3)	182 (12.4)	127 (8.3)	178 (7.5)
		3 year	440 (11.1)	152 (12.1)	288 (10.7)	258 (17.5)	187 (12.2)	253 (10.6)
FFR group	*N* (%)		1,981	629 (31.8)	1,352 (68.2)	736 (37.2)	864 (43.6)	1,117 (56.4)
	Cumulative mortality *N* (%)	1 year	65 (3.3)	23 (3.7)	42 (3.1)	43 (5.8)	35 (4.1)	30 (2.7)
		2 year	128 (6.5)	45 (7.2)	83 (6.1)	73 (9.9)	64 (7.4)	64 (5.7)
		3 year	190 (9.6)	64 (10.2)	126 (9.3)	110 (14.9)	93 (10.8)	97 (8.7)
Angio-graphy-only group	*N* (%)		1,981	629 (31.8)	1,352 (68.2)	735 (37.1)	711 (35.9)	1,270 (64.1)
	Cumulative mortality *N* (%)	1 year	97 (4.9)	31 (4.9)	66 (4.9)	66 (9.0)	32 (4.5)	65 (5.1)
		2 year	177 (8.9)	63 (10.0)	114 (8.4)	109 (14.8)	63 (8.9)	114 (9.0)
		3 year	250 (12.6)	88 (14.0)	162 (12.0)	148 (20.1)	94 (13.2)	156 (12.3)
Relative risk reduction FFR vs. angiography-only group % (95%-CI)			24% (9.2%–36.4%)	27% (1.6%–46.3%)	22% (3.1%-37.6%)	26% (7.1%–40.7%)	19% 6.5%–37.8%)	29% (10.1%–44.4%)
NNT to prevent one death at 3 years for FFR vs. angiography-only guidance (95%-CI)			34 (20–94)	27 (14–456)	38 (21–295)	20 (12–77)	41 (18–∞)	28 (17–89)

FFR, means fractional flow reserve; PCI, percutaneous coronary intervention; OMT, optimal medical therapy; ACS, acute coronary syndrome; CCS, chronic coronary syndrome; y, follow-up year; and NNT, number needed to treat.

**Figure 4:**
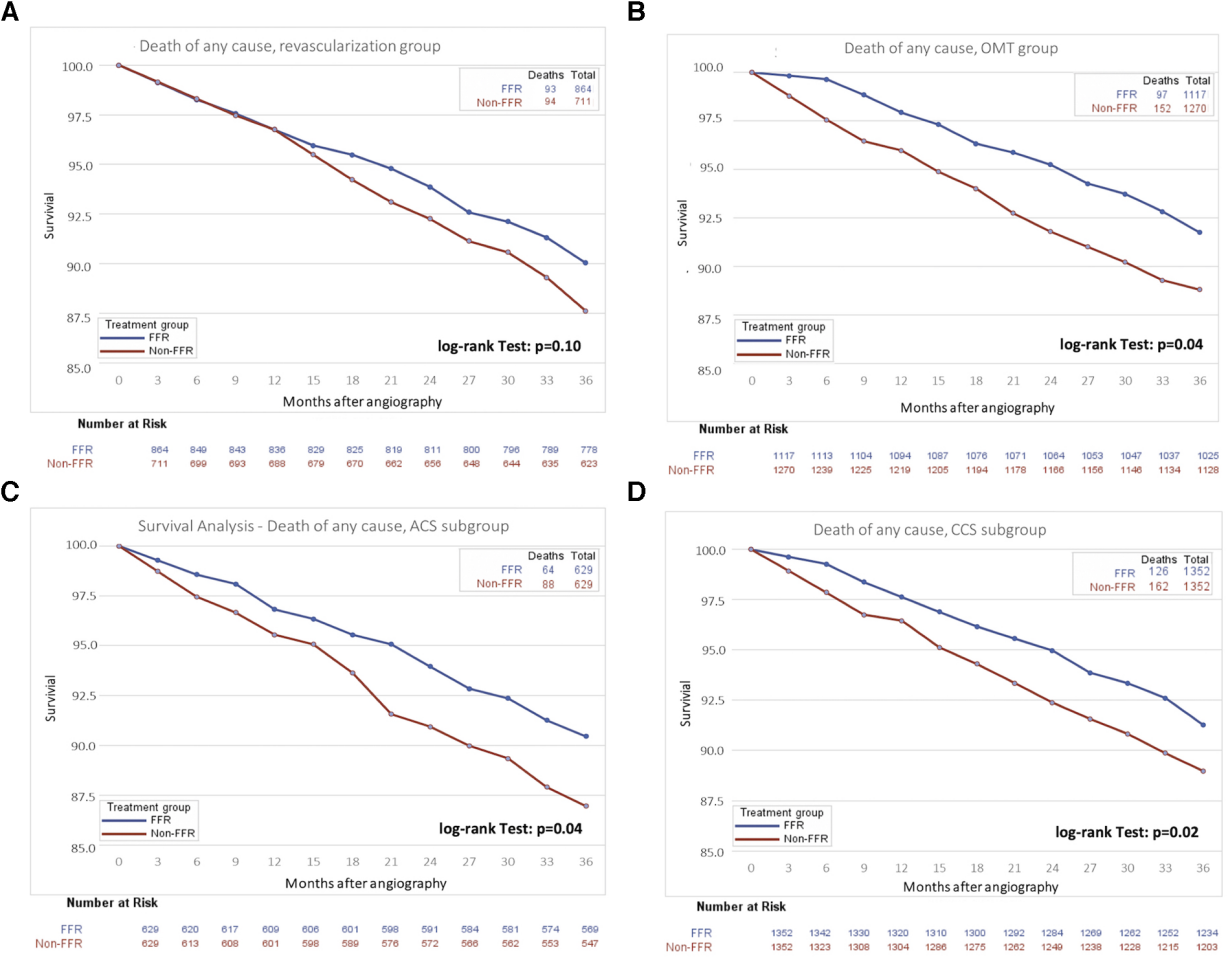
Survival curves for survival in subgroups of patients undergoing FFR or angiography guidance for coronary artery disease therapy. (**A**) Patients undergoing coronary revascularization by either PCI or CABG, (**B**) Patients undergoing optimal medical therapy, (**C**) patients presenting with ACS, and (**D**) patients presenting with CCS. ACS, acute coronary syndrome; CCS, chronic coronary syndrome; FFR, fractional flow reserve; OMT, optimal medical therapy.

In patients undergoing PCI, mortality rates were lower in the group guided by FFR compared with those treated with angiographic guidance alone (10.8% vs. 13.2%; *p* = 0.10). Similarly, in patients undergoing conservative management with OMT, mortality rates were lower in the FFR as compared with the angiography-only group (8.7% vs. 12.3%; *p* = 0.04, Figure 4). Of note, in those patients managed conservatively with OMT, the reduction in mortality observed with FFR was evident immediately after treatment initiation (Figure 4B), whereas in the revascularization group, this effect was observed after 12 months (Figure 4A).

An FFR-guided treatment strategy was associated with a lower 3-year mortality in the subgroup of patients aged ≥75 years (14.9% vs. 20.1%, *p* < 0.001), in patients presenting with ACS (10.2% vs. 14.0%, *p* = 0.04, Table 3, Figure 4C), and those presenting with CCS (9.3% vs. 12.0%, *p* = 0.02, Figure 4D).

## Discussion

The entire FLORIDA study examines the impact of FFR guidance for coronary revascularization on mortality using a large, contemporary cohort of 64,045 patients undergoing coronary angiography. Its results add to the evidence to support an FFR-based strategy for coronary revascularization, as using FFR to decide on treatment strategy was associated with a significantly reduced mortality at 3 years compared with angiographic-based decision. Importantly, mortality benefits for the FFR group were even more pronounced in the high-risk subgroups of elderly patients and those presenting with ACS. Hence, our study clearly supports the current European (4) as well as American guideline recommendations (5) for functional lesion interrogation by FFR, when presence of ischemia on non-invasive imaging is not available, and provides additional evidence on the use of FFR especially in older patients and patients presenting with ACS.

### FFR and mortality benefits in real-world patients

The present study provides a comprehensive analysis of the association of the use of FFR to guide revascularization/treatment with mortality in a nationwide, large, unselected contemporary cohort of patients with suspected coronary artery disease.

Observational data investigating FFR in real-life settings are conflicting. While some analyses do not show any benefits for FFR use (17), superior outcomes with respect to myocardial infarction and repeat revascularization were observed after routine use of FFR in a single-center study (18). More recent data from the Swedish Coronary Angiography Registry (SCAAR) demonstrated a 20% reduction in mortality at 5 years with FFR use but included also patients treated with bare metal stents, who were therefore at increased risk of complications (11). Further, an electronic health records analysis from the Veterans Affairs Clinical Assessment, Reporting, and Tracking (VA CART) Program reported a 43% reduction in 1-year mortality using FFR measurement (10). This more pronounced reduction in mortality may be due to the overall increased baseline risk in the VA CART study population, with over half of patients being diabetic, and a variable use of non-invasive imaging among health care systems may also come into play. FLORIDA extends these findings to patients treated in overwhelming majority with new generation drug-eluting stents, since bare metal stents are only used sporadically in Germany, and to the entire spectrum of coronary artery disease, including patients with ACS and varying degrees of coronary artery disease severity, thereby complementing existing randomized clinical trial data (8, 19).

Although no other endpoints were evaluated, we hypothesize that the survival benefit in patients undergoing FFR-guided PCI in our study might result from a reduced risk of myocardial infarction in the FFR group, since the relationship between use of FFR guidance and lower risk of myocardial infarction has been shown previously (11, 20), and a survival benefit after myocardial revascularization is closely related to a reduction in myocardial infarction (21).

All randomized trials in this area have failed to demonstrate mortality benefits for several reasons: studies were often underpowered since they included small cohorts of chronic coronary artery disease patients at only low- to intermediate risk. The population of FLORIDA represents an all-comer patient population at a mean age of 68.6 years, with 37% being female, 33% diabetic, and 37% presenting with ACS as index event. These baseline characteristics contrast, for example, with the younger, lower-risk FAME population that included only patients with unstable angina as a selected ACS subgroup (6). This may also have caused the slightly higher proportion of patients treated by revascularization therapy in the FFR-group (43.6%) compared to the angiography–only group (35.9%) as observed in FLORIDA, which is in contrast to randomized studies, where at least the number of implanted stents was reduced using FFR (6).

While mortality in FAME was 3.2% at 2 years (22) and 9.3% at 5 years (23), and 2.2% at 2 years (24) and 7.1% (25) at 5 years in DEFER, mortality in FLORIDA was 7.7% at 2 years and 11.1% at 3 years. Thus, the FLORIDA population showed a considerably higher mortality than the study populations in FAME or DEFER, confirming the adverse risk profile in our study population. Higher rates of mortality in observational studies compared with clinical trials have previously been reported (26, 27), and may be at least in part due to the highly selected patient populations enrolled in randomized trials and the real-world conditions including community and tertiary centers, along with substantial variation in physicians' practice (28, 29). This higher mortality observed in real-world settings has the inherent advantage of an increased statistical power for the detection of mortality differences among groups.

When subjecting patients to the type of treatment, i.e., coronary revascularization or conservative management with OMT, following hemodynamic lesion assessment with FFR, FLORIDA provides insights into the temporal course of FFR-related benefits. While early benefits of FFR guidance were observed in patients undergoing conservative management, a trend towards long-term benefits of FFR guidance was also observed in patients undergoing coronary revascularization with numerical mortality differences emerging after 1 year. Since previous studies demonstrated over- or underestimation of coronary lesions using angiographic guidance (30, 31), FFR guidance allowed improved identification of stenotic coronary lesions with or without functional significance (3). Therefore, early benefits of FFR-based deferral of revascularization observed in our study might be attributed to a reduction of unnecessary revascularization compared to isolated angiographic guidance (6) thus potentially reducing the early risk of bleeding and stent-related complications.

In contrast, using angiography only may have resulted in (unnecessary) PCI in patients that were falsely subjected to revascularization and might not have been treated in the FFR-guided group, which may account for the long-term benefit at 1 year in the FFR group undergoing revascularization. Indeed, Zimmermann et al. reported an increased risk of late myocardial infarctions in such lesions that were formerly stented, although being classified as functionally non-significant by FFR (20). In support of this findings, analysis of the SCAAR (Swedish Coronary Angiography and Angioplasty Registry) (11) also revealed a long-term benefit favoring FFR-based revascularization with divergence of the survival curves after 2-years which is comparable to the (late) survival benefit in our study. These observations are important, particularly in view of the recently-published ISCHEMIA trial (32). ISCHEMIA did not show any prognostic benefit for revascularization therapy compared with medical management in patients with CCS and evidence of moderate or severe myocardial ischemia on non-invasive testing within 3 years. With respect to the time course of clinical benefits related to FFR-guided treatment observed in FLORIDA, a longer observational period as well as the use of FFR for PCI guidance may have resulted in different conclusions from ISCHEMIA, although these considerations remain speculative.

### Potential of FFR to guide coronary revascularization in the elderly

In FLORIDA, benefits of FFR were most pronounced in high-risk patients including elderly patients and those presenting with ACS, in whom avoiding any risk of bleeding and stent-related complications when PCI is deferred based on FFR results might be particularly beneficial (33). Indeed, a third of patients in FLORIDA were older than 75 years of age and had a substantial burden of comorbidities as reflected by a high need for anti-inflammatory or corticosteroid co-medication. Within the well-matched FLORIDA patient cohort with age used as separate matching factor, elderly patients over 75 years of age had a strong benefit with a 26% relative risk reduction and an NNT of 20 to prevent one death. These findings clearly support the use of FFR in every-day clinical practice, and particularly in high-risk, elderly patients (34, 35).

Importantly, despite the increased complexity of patients in this study compared with randomized trials, the proportion of patients undergoing coronary revascularization was substantially lower than those reported in randomized trials; for example, 38% patients included in FLORIDA received coronary revascularization in comparison to 72% in DEFER (24).

### Potential of FFR to guide coronary revascularization in ACS

FFR-guided coronary revascularization in ACS patients remains ill-defined (36). Whereas FAMOUS-NSTEMI revealed no significant differences in mortality between the strategies considered (16, 36), a large retrospective analysis from the US-National Readmissions Data reported lower rates of in-hospital mortality for patients presenting with ACS undergoing FFR-guidance during PCI (37). In FLORIDA, with ACS used as separate matching factor, ACS patients had the strongest benefit with a 27% relative risk reduction and an NNT of 27, which is considered a substantial effect for a diagnostic methodology; future sufficiently-powered randomized trials are warranted to confirm these results.

Given the resistance for FFR utilization in every-day clinical practice observed in this study and others (10), FLORIDA adds further arguments to consequently respect FFR as an evidence-based, diagnostic, and prognostically meaningful tool for treatment and revascularization guidance, which can be expanded to the entire spectrum of patients with coronary artery disease, including particularly elderly patients and those presenting with ACS.

### Study limitations

Retrospective analyses of insurance data for the association of different treatments with mortality have inherent limitations (38), even when using robust statistical methodology to minimize confounding. First, our dataset did not comprise information on education level, socio-demographic status, lifestyle behavior (e.g., smoking, and physical activity), multivessel-disease and angiographic lesion severity, particularly with respect to the lesions examined with FFR. Although we carefully adjusted the two groups with a mix of direct and PS matching including 72 variables to yield a balanced distribution of baseline characteristics between the two comparator groups to minimize confounding, residual confounding cannot be entirely excluded due to the retrospective nature of this study. Second, as information on the cause of death was not available, analyses of cardiovascular and non-cardiovascular mortality were precluded. Third, as the database represents a health claims database, the possibility of misclassification and miscoding of data cannot be ruled out completely and adjudication of events was not possible. However, as structured insurance data were used, any selection or reporting bias can be excluded. Fourth, although a relative low penetration of FFR use (compared to previous large studies) needs to be taken into account when interpreting the results of this study, we provide additional evidence beyond recently published data as mortality benefits for the FFR group in our study were even more pronounced in the high-risk subgroups of elderly patients and those presenting with ACS in whom a routine physiological assessment of coronary lesions is less well investigated (4, 5) and former studies emphasized that elderly (unfortunately) have lower odds of receiving FFR guidance (39). Finally, the penetration of FFR also depends on geographical or regional aspects and the overall frequency of FFR usage in Germany previously reported by others is in line with our study (40).

## Conclusions

This observational study demonstrates a lower long-term mortality using FFR compared to angiographic guidance to determine treatment strategy in patients undergoing coronary angiography for suspected coronary artery disease, using an all-comers approach and a matching design. Mortality benefits of FFR were robust, irrespective of whether PCI was performed or not, and were more pronounced in elderly patients and those presenting with ACS. The results of FLORIDA strongly support current guideline recommendations and promote the use of FFR for decision-making in coronary artery disease therapy.

### Impact on daily practice

FFR is a validated, guideline-based diagnostic tool for hemodynamic coronary lesion assessment of coronary lesions and PCI guidance. Limited data exist regarding differences in long-term mortality between FFR-guided and angiographic-guided treatment strategies, mainly because clinical trials were not sufficiently powered to show differences in mortality. The observational FLORIDA study included a large, unselected all-comer patient cohort and showed a 24% lower 3-year mortality in patients undergoing FFR as compared to those undergoing angiographic-guidance for advanced CAD. These benefits were more pronounced in high-risk subgroups including aged and ACS patients.

## Data Availability

The raw data supporting the conclusions of this article will be made available by the authors, without undue reservation.
