# Supplementary material for: Fractional flow reserve measurements and long-term mortality—results from the FLORIDA study
Source: Front Cardiovasc Med. 2024 Feb 9;11:1337941. doi: 10.3389/fcvm.2024.1337941 (PMC10885355; doi:10.3389/fcvm.2024.1337941)
Supplement: Supplementary file 2 [file Table2.docx]

**Boeckling et. al.: Supplementary Table S1. Predictors of FFR Utilization from propensity score estimation**

|  | **ATC/ICD/ICPM/ EBM-Code** | **Odds Ratio**  **(95%-Confidence Interval)** | **p-value** |
| --- | --- | --- | --- |
| **Medication** | | | |
| *Beta blocking agents* | | | |
| Beta blocking agents, plain, selective | C07AB | **1.176 (1.061 – 1.303)** | **0.0021** |
| Beta blocking agents, others | C07AA, C07AG, C07BA, C07BB, C07BG, C07CA, C07CB, C07CG, C07DA, C07DB, C07EA, C07EB, C07FB, C07FX | 0.93 (0.586 **–** 1.474) | 0.7563 |
| *Calcium antagonists* |  |  |  |
| Dihydropyridine derivatives | C08CA | 1.008 (0.784 **–** 1.297) | 0.9484 |
| Calcium antagonists, others | C08CX, C08DA, C08DB, C08EA,  C08EX , C08GA | 1.008 (0.791 **–** 1.286) | 0.9457 |
| *ACE inhibitor+ARB* |  |  |  |
| ACE inhibitors, plain | C09AA | 0.867 (0.697 **–** 1.078) | 0.1995 |
| ACE inhibitors and diuretics | C09BA | 0.987 (0.855 **–** 1.138) | 0.8539 |
| Angiotensin II antagonists, plain | C09CA | 1.077 (0.946 **–** 1.226) | 0.2639 |
| Angiotensin II antagonists and diuretics | C09DA | 1.013 (0.867 **–** 1.183) | 0.8722 |
| ACE inhibitor+ARB, others | C09BB, C09BX, C09DB, C09DX | 1.093 (0.886 **–** 1.349) | 0.4078 |
| *Statins* |  |  |  |
| HMG COA reductase inhibitors | C10AA | 1.135 (0.891 **–** 1.447) | 0.3046 |
| Statins, others | C10BA, C10BX | 1.156 (0.902 **–** 1.483) | 0.2521 |
| *Anti-platelet therapy* |  |  |  |
| Platelet aggregation inhibitors excluding heparin | B01AC | 0.826 (0.671 **–** 1.017) | 0.0724 |
| Thrombin inhibitors, others | B01AE, B01AF, B01AX | **1.32 (1.069 – 1.630)** | **0.0099** |
| Nitrates | C01DA, C01DX, C01EB18 | 1.069 (0.832 **–** 1.373) | 0.6004 |
| Aspirin | N02BA01, A01AD05, B01AC06 | 0.871 (0.526 **–** 1.442) | 0.5919 |
| Clopidogrel | B01AC04, B01AC34 | 0.924 (0.773 **–** 1.104) | 0.3834 |
| Ticagrelor and prasugrel | B01AC24, B01AC22 | 0.854 (0.600 **–** 1.215) | 0.3797 |
| Vitamin K antagonists plus NOACS | B01AA, B01AF02, B01AF01, B01AF03, B01AE07 | **0.815 (0.688 – 0.966)** | **0.0181** |
| Isosorbide mononitrate | C01DA14, C01DA58 | 0.794 (0.554 **–** 1.138) | 0.2092 |
| Fibrates | C10AB | 1.03 (0.702 **–** 1.513) | 0.8785 |
| *Other common comedication* |  |  |  |
| Alpha-adrenoreceptor antagonists | G04CA | 1.089 (0.919 **–** 1.29) | 0.3241 |
| Glucocorticoids | H02AB | **0.828 (0.715 – 0.959)** | **0.0117** |
| Thyroid hormones | H03AA | 0.992 (0.880 **–** 1.119) | 0.8968 |
| Fluoroquinolones | J01MA | 1.018 (0.889 **–** 1.166) | 0.7966 |
| Acetic acid derivatives and related substances | M01AB | 0.898 (0.789 **–** 1.022) | 0.1044 |
| Propionic acid derivatives | M01AE | 1.036 (0.932 **–** 1.151) | 0.5174 |
| Preparations inhibiting uric acid production | M04AA | 0.973 (0.848 **–** 1.116) | 0.6953 |
| Pyrazolones | N02BB | 1.02 (0.911 **–** 1.142) | 0.7277 |
| Proton pump inhibitors | A02BC | **1.118 (1.014 – 1.234)** | **0.0258** |
| Biguanides | A10BA | 1.085 (0.929 **–** 1.268) | 0.3006 |
| Sulfonamides, plain | C03BA | **0.783 (0.685 – 0.895)** | **0.0003** |
| **Diseases** |  |  |  |
| *Cardio-vascular diseases* |  |  |  |
| Chronic ischemic heart disease | I25 | **1.38 (1.227 – 1.553)** | **<0.0001** |
| Previous myocardial infarction - existing | I25.2 | 0.989 (0.864 **–** 1.133) | 0.8730 |
| Heart failure – NYHA I or unknown | I50.11, I50.9 | 0.842 (0.674 **–** 1.051) | 0.1282 |
| Heart failure – NYHA II or III | I50.12, I50.13 | **0.800 (0.651 – 0.983)** | **0.0333** |
| Heart failure – NYHA IV | I50.14 | 0.869 (0.650 **–** 1.163) | 0.3460 |
| Other cardiac arrhythmias | I49 | 1.038 (0.923 **–** 1.167) | 0.5351 |
| Previous stroke - acute | I21, I22 | 0.842 (0.695 **–** 1.020) | 0.0787 |
| Previous stroke - existing | I69 | 1.137 (0.900 **–** 1.438) | 0.2819 |
| Presence of cardiac and vascular implants and grafts | Z95 | **0.803 (0.707 – 0.911)** | **0.0007** |
| *Diabetes* |  |  |  |
| Type 2 diabetes mellitus | E11 | **0.699 (0.577 – 0.847)** | **0.0003** |
| Other diabetes mellitus | E10, E13, E14 | 1.19 (0.980 **–** 1.446) | 0.0797 |
| *Other cardio-vascular risk factors* |  |  |  |
| Essential (primary) hypertension | I11 | 1.022 (0.781 **–** 1.338) | 0.8747 |
| Disorders of lipoprotein metabolism and other lipidemias | E78.1 – E78.9 | 1.069 (0.889 **–** 1.285) | 0.4775 |
| Kidney failure | N19 | 1.127 (0.823 **–** 1.542) | 0.4557 |
| Hypercholesterolemia | E78.0 | 0.95 (0.787 **–** 1.147) | 0.5932 |
| Pulmonary Hypertension | I27 | 1.209 (0.904 **–** 1.618) | 0.2010 |
| Overweight and obesity | E66 | **1.36 (1.031 – 1.794)** | **0.0294** |
| *Other diseases* |  |  |  |
| Spondylosis | M47 | **1.155 (1.033 – 1.291)** | **0.0112** |
| Thoracic, thoracolum, and lumbosacral intvrt disc disorders | M51 | 1.069 (0.947 **–** 1.206) | 0.2801 |
| Dorsalgia | M54 | 1.011 (0.915 **–** 1.117) | 0.8338 |
| Hyperplasia of prostate | N40 | **1.159 (1.019 – 1.318)** | **0.0248** |
| Encounter for screening for malignant neoplasms | Z12 | 1.038 (0.935 **–** 1.152) | 0.4841 |
| Disorders of refraction and accommodation | H52 | 1.06 (0.960 **–** 1.170) | 0.2504 |
| Osteoarthritis of knee | M17 | **0.861 (0.764 – 0.969)** | **0.0135** |
| Presence of other functional implants | Z96 | 1.017 (0.904 **–** 1.145) | 0.7756 |
| **Previous interventions** | | | |
| Creation of an aortocoronary bypass | 5-361 | **0.121 (0.030 – 0.488)** | **0.0030** |
| Creation of an aortocoronary bypass by minimally invasive technique (Inpatient) | 5-362 | 0.325 (0.045 **–** 2.372) | 0.2680 |
| Inpatient angiocardiography | 1-276.0 | 0.438 (0.107 **–** 1.787) | 0.2501 |
| Other diagnostic catheter examination of the heart and vessels (inpatient) | 1-279 | **2.43 (1.573 – 3.754)** | **<0.0001** |
| Other outpatient intervention (PTCA, Stent) | 34286 | **0.281 (0.088 – 0.900)** | **0.0326** |
| Outpatient serial angiogram | 34284, 34283 | 1.18 (0.601 **–** 2.316) | 0.6308 |
| Placement of a drug-eluting bifurcation stent (inpatient) | 8-837.v | 0.22 (0.030 **–** 1.608) | 0.1356 |
| Transarterial left heart catheter examination (inpatient) | 1-275 | **1.31 (1.065 – 1.611)** | **0.0105** |
| Transarterial left heart catheter examination (outpatient) | 34291 | **1.728 (1.235 – 2.417)** | **0.0014** |
| *Previous stents in a coronary artery* | 8-837.k, 8-837.m |  |  |
| 1 stent |  | 1.228 (0.881 **–** 1.710) | 0.2254 |
| 2 stents |  | 0.856 (0.552 **–** 1.327) | 0.4859 |
| 2 stents in several coronary arteries |  | 0.751 (0.337 **–** 1.675) | 0.4846 |
| 3 stents |  | 1.317 (0.746 **–** 2.324) | 0.3421 |
| 3 stents in several coronary arteries |  | 0.946 (0.370 **–** 2.416) | 0.9077 |
| 4 stents |  | 0.739 (0.225 **–** 2.422) | 0.6173 |
| 4 stents in several coronary arteries |  | 0.419 (0.057 **–** 3.113) | 0.3956 |

Statistically significant predictors to α=0.05 are marked bold. Disease names correspond to the translation of ICD-10-GM codes, medication names to the description of the ATC classification and procedures to the translation of the German ICPM and EBM codes.

Abbreviations: EBM means Einheitlicher Bewertungsmaßstab, *Doctors' fee scale,* ICD-10-GM = International statistical classification of diseases and related health problems, 10^th^ revision, German modification, and ICPM = International classification of procedures in medicine

**Boeckling et. al.: Supplementary Table S2. Baseline Diagnosis and Medication Characteristics before Matching**

|  | **Before Matching** | | | |
| --- | --- | --- | --- | --- |
|  | **Total study cohort** | **Angiography + FFR** | **Angiography only** | **SMD** |
|  |  |  |  |  |
| **N** | 64,045 | 1,992 | 62,053 | -- |
| **Most common comorbidities as ICD-10 codes** | | | | |
| *Cardio-vascular diseases* |  | | | |
| I25 -Chronic ischemic heart disease | 29,773 (46.5) | 1,160 (58.2) | 28,613 (46.1) | 24.45 |
| I50 -Congestive heart failure | 14,479 (22.6) | 426 (21.4) | 14,053 (22.6) | -3.04 |
| I49 -Ventricular fibrillation and flutter | 11,810 (18.4) | 398 (20.0) | 11,412 (18.4) | 4.04 |
| I48 -Atrial flutter | 11,132 (17.4) | 332 (16.7) | 10,800 (17.4) | -1.96 |
| *Diabetes mellitus including:* |  | | | |
| E11 -Type 2 diabetes mellitus | 21,124 (33.0) | 645 (32.4) | 20,479 (33.0) | -1.33 |
| E14 –Unspecified diabetes mellitus | 13,126 (20.5) | 443 (22.2) | 12,683 (20.4) | 4.39 |
| *Other cardio-vascular risk factors* |  | | | |
| I10 -Essential (primary) hypertension | 49,509 (77.3) | 1,653 (83.0) | 47,856 (77.1) | 14.71 |
| E78 -Disorders of lipoprotein metabolism and other lipidaemias | 37,380 (58.4) | 1,295 (65.0) | 36,085 (58.2) | 14.13 |
| E66 -Obesity | 14,853 (23.2) | 481 (24.1) | 14,372 (23.2) | 2.32 |
| N19 -Kidney failure | 990 (1.5) | 37 (1.9) | 953 (1.5) | 2.49 |
| *Other diseases* |  | | | |
| M54 -Dorsalgia | 28,164 (44.0) | 927 (46.5) | 27,237 (43.9) | 5.31 |
| M51 -Other intervertebral disc disorder | 11,291 (17.6) | 402 (20.2) | 10,889 (17.5) | 6.73 |
| M47 -Spondylosis | 14,708 (23.0) | 534 (26.8) | 14,174 (22.8) | 9.19 |
| N40 -Hyperplasia of prostate | 11,989 (18.7) | 457 (22.9) | 11,532 (18.6) | 10.76 |
| H52 -Disorders of refraction and accommodation | 24,325 (38.0) | 813 (40.8) | 23,512 (37.9) | 5.99 |
| M17 -Gonarthrosis [arthrosis of knee] | 13,256 (20.7) | 388 (19.5) | 12,868 (20.7) | -3.14 |
| **Most common comedications** | | | | |
| M01AE -NSAIDs | 16,338 (25.5) | 533 (26.8) | 15,805 (25.5) | 2.93 |
| A02BC -Proton pump inhibitors | 25,647 (40.0) | 888 (44.6) | 24,759 (39.9) | 9.48 |
| B01AC -Platelet aggregation inhibitors excl. heparin | 14,633 (22.8) | 588 (29.5) | 14,045 (22.6) | 15.73 |
| A10BA -Biguanides | 8,694 (13.6) | 290 (14.6) | 8,404 (13.5) | 2.92 |
| J01MA -Fluoroquinolones | 8,052 (12.6) | 260 (13.1) | 7,792 (12.6) | 1.48 |
| G04CA -Alpha-adrenoreceptor antagonists | 5,543 (8.7) | 218 (10.9) | 5,325 (8.6) | 7.97 |
| C03CA -Sulfonamides, plain | 14,339 (22.4) | 384 (19.3) | 13,955 (22.5) | -7.91 |
| M01AB -Acetic acid derivatives and related substances | 10,003 (15.6) | 293 (14.7) | 9,710 (15.6) | -2.62 |
| **ACE inhibitors/ARB** |  | | | |
| C09BA -ACE inhibitors and diuretics | 7,443 (11.6) | 245 (12.3) | 7,198 (11.6) | 2.16 |
| C09DA -Angiotensin II receptor blockers (ARBs) and diuretics | 6,192 (9.7) | 215 (10.8) | 5,977 (9.6) | 3.84 |
| C09AA -ACE inhibitors, plain | 21,604 (33.7) | 706 (35.4) | 20,898 (33.7) | 3.71 |
| C09CA -Angiotensin II receptor blockers (ARBs), plain | 9,727 (15.2) | 352 (17.7) | 9,375 (15.1) | 6.93 |
| C07AB -Beta blocking agents, selective | 30,944 (48.3) | 1,122 (56.3) | 29,822 (48.1) | 16.61 |
| H03AA -Thyroid hormones | 11,231 (17.5) | 351 (17.6) | 10,880 (17.5) | 0.23 |
| M04AA -Preparations inhibiting uric acid production | 8,807 (13.8) | 271 (13.6) | 8,536 (13.8) | -0.44 |
| H02AB -Glucocorticoids | 7,977 (12.5) | 218 (10.9) | 7,759 (12.5) | -4.85 |
| N02BB -Pyrazolones | 14,728 (23.0) | 480 (24.1) | 14,248 (23.0) | 2.68 |
| C10AA -HMG CoA reductase inhibitors | 25,752 (40.2) | 1,019 (51.2) | 24,733 (39.9) | 22.83 |
| C08CA -Dihydropyridine derivatives | 14,791 (23.1) | 500 (25.1) | 14,291 (23.0) | 4.84 |

Values are n (%). Angiography-only group received angiography without FFR guidance. SMD is in %. Disease names correspond to the translation of ICD-10-GM codes.

Abbreviations: ACE means angiotensin-converting-enzyme, ARBs = Angiotensin II receptor blockers, FFR = Fractional flow reserve, HMG CoA = β-Hydroxy β-methylglutaryl- coenzyme A, NSAID = Non-steroidal anti-inflammatory drug, and SMD = Standardized mean difference.

**Boeckling et. al.: Supplementary Table S3. Baseline Diagnosis and Medication Characteristics after Matching**

|  | **After Matching** | | | |
| --- | --- | --- | --- | --- |
|  | **Total study cohort** | **Angiography + FFR** | **Angiography only** | **SMD**  **(p-value)** |
| **N** | 3,962 | 1,981 | 1,981 | --- |
| **Most common comorbidities as three-digit ICD-10 codes** | | | | |
| *Cardio-vascular diseases* |  | | | |
| I25 -Chronic ischemic heart disease | 2,301 (58.1) | 1,149 (58.0) | 1,152 (58.2) | -0.31 (0.9487) |
| I50 -Congestive heart failure | 864 (21.8) | 425 (21.5) | 439  (22.2) | -1.71 (0.6170) |
| I49 -Ventricular fibrillation and flutter | 795 (20.1) | 396 (20.0) | 399 (20.1) | -0.38 (0.9368) |
| I48 -Atrial flutter | 687 (17.3) | 330 (16.7) | 357  (18.0) | -3.6 (0.2752) |
| *Diabetes mellitus including:* |  | | | |
| E11 -Type 2 diabetes mellitus | 1,273 (32.1) | 643 (32.5) | 630  (31.8) | 1.41 (0.6831) |
| E14 –Unspecified diabetes mellitus | 883 (22.3) | 441 (22.3) | 442 (22.3) | -0.12 (>0.9999) |
| *Other cardio-vascular risk factors* |  | | | |
| I10 -Essential (primary) hypertension | 3,271 (82.6) | 1,642 (82.9) | 1,629 (82.2) | 1.73 (0.6154) |
| E78 -Disorders of lipoprotein metabolism and other lipidaemias | 2,625 (66.3) | 1,287 (65.0) | 1,338 (67.5) | -5.45 (0.0929) |
| E66 -Obesity | 938 (23.7) | 478 (24.1) | 460  (23.2) | 2.14 (0.5252) |
| N19 -Kidney failure | 65 (1.6) | 37 (1.9) | 28  (1.4) | 3.58 (0.3171) |
| *Other diseases* |  |  |  |  |
| M54 -Dorsalgia | 1,839 (46.4) | 923 (46.6) | 916 (46.2) | 0.71 (0.8484) |
| M51 -Other intervertebral disc disorder | 792 (20.0) | 399 (20.1) | 393 (19.8) | 0.76 (0.8426) |
| M47 -Spondylosis | 1,050 (26.5) | 531 (26.8) | 519  (26.2) | 1.37 (0.6921) |
| N40 -Hyperplasia of prostate | 895 (22.6) | 455 (23.0) | 440  (22.2) | 1.81 (0.5948) |
| H52 -Disorders of refraction and accommodation | 1,592 (40.2) | 810 (40.9) | 782  (39.5) | 2.88 (0.3816) |
| M17 -Gonarthrosis [arthrosis of knee] | 735 (18.6) | 386 (19.5) | 349  (17.6) | 4.81 (0.1412) |
| **Most common comedications** | | | | |
| M01AE -NSAIDs | 1,057 (26.7) | 531 (26.8) | 526  (26.6) | 0.57 (0.8858) |
| A02BC -Proton pump inhibitors | 1,756 (44.3) | 881 (44.5) | 875 (44.2) | 0.61 (0.8730) |
| B01AC -Platelet aggregation inhibitors excl. heparin | 1,162 (29.3) | 578 (29.2) | 584  (29.5) | -0.67 (0.8615) |
| A10BA -Biguanides | 571 (14.4) | 288 (14.5) | 283  (14.3) | 0.72 (0.8564) |
| J01MA -Fluoroquinolones | 513 (12.9) | 259 (13.1) | 254  (12.8) | 0.75 (0.8499) |
| C09CA -Angiotensin II receptor blockers (ARBs), plain | 690 (17.4) | 348 (17.6) | 342  (17.3) | 0.8 (0.8341) |
| G04CA -Alpha-adrenoreceptor antagonists | 427 (10.8) | 217 (11.0) | 210  (10.6) | 1.14 (0.7586) |
| C03CA -Sulfonamides, plain | 773 (19.5) | 382 (19.3) | 391  (19.7) | -1.15 (0.7484) |
| M01AB -Acetic acid derivatives and related substances | 593 (15.0) | 292 (14.7) | 301  (15.2) | -1.27 (0.7217) |
| C09BA -ACE inhibitors and diuretics | 476 (12.0) | 244 (12.3) | 232  (11.7) | 1.86 (0.5910) |
| C07AB -Beta blocking agents, selective | 2,209 (55.8) | 1,114 (56.2) | 1,095 (55.3) | 1.93 (0.5648) |
| H03AA -Thyroid hormones | 718 (18.1) | 351 (17.7) | 367  (18.5) | -2.1 (0.5362) |
| C09DA -Angiotensin II receptor blockers (ARBs) and diuretics | 414 (10.4) | 214 (10.8) | 200  (10.1) | 2.31 (0.4996) |
| M04AA -Preparations inhibiting uric acid production | 556 (14.0) | 269 (13.6) | 287  (14.5) | -2.62 (0.4368) |
| C09AA -ACE inhibitors, plain | 1,425 (36.0) | 700 (35.3) | 725  (36.6) | -2.63 (0.4269) |
| H02AB -Glucocorticoids | 457 (11.5) | 218 (11.0) | 239  (12.1) | -3.32 (0.3199) |
| N02BB -Pyrazolones | 925 (23.3) | 477 (24.1) | 448  (22.6) | 3.46 (0.2930) |
| C10AA -HMG CoA reductase inhibitors | 2,064 (52.1) | 1,011 (51.0) | 1,053 (53.2) | -4.24 (0.1923) |
| C08CA -Dihydropyridine derivatives | 953 (24.1) | 495 (25.0) | 458  (23.1) | 4.37 (0.1808) |

Values are n (%). Angiography-only group received angiography without FFR guidance. SMD is in %. Disease names correspond to the translation of ICD-10-GM codes.

Abbreviations: ACE means angiotensin-converting-enzyme, ARBs = Angiotensin II receptor blockers, FFR = Fractional flow reserve, HMG CoA = β-Hydroxy β-methylglutaryl- coenzyme A, NSAID = Non-steroidal anti-inflammatory drug, and SMD = Standardized mean difference.
